# Goal-directed control with cortical units that are gated by both top-down feedback and oscillatory coherence

**DOI:** 10.1186/1471-2202-15-S1-P197

**Published:** 2014-07-21

**Authors:** Robert R Kerr, David B Grayden, Doreen A Thomas, Matthieu Gilson, Anthony N Burkitt

**Affiliations:** 1 NeuroEngineering Laboratory, Dept. of Electrical & Electronic Engineering, University of Melbourne, Australia; 2 Centre for Neural Engineering, University of Melbourne, Australia; 3 NICTA, Victoria Research Lab, University of Melbourne, Australia; 4 Bionics Institute, East Melbourne, Australia; 5 Department of Mechanical Engineering, University of Melbourne, Australia; 6 Laboratory for Neural Circuit Theory, RIKEN Brain Science Institute, Saitama, Japan; 7 Computational Neuroscience Group, University Pompeu Fabra, Barcelona, Spain

## 

The brain is able to flexibly select behaviors that adapt to both its environment and its present goals. This cognitive control is understood to occur within the hierarchy of the cortex and relies strongly on the prefrontal and premotor cortices [1], which sit at the top of this hierarchy. Pyramidal neurons, the principal neurons that form the basis of the functional circuits in the cortex, have been observed to exhibit much stronger responses when they receive inputs at their soma/basal dendrites that are coincident with inputs at their apical dendrites [2]. This corresponds to receiving inputs simultaneously from both higher-order regions (feedback) and lower-order regions (feedforward) [3]. In addition to this, temporal coherence between oscillations, such as gamma oscillations, in different neuronal groups has been proposed to modulate and route communication in the brain [4].

In this study, we develop a simple, but novel, neural mass model in which cortical units (or ensembles) of pyramidal neurons and inhibitory interneurons exhibit gamma oscillations when they receive coherent oscillatory inputs from both feedforward and feedback connections. In this way, the activity of these units is gated by both top-down feedback and oscillatory coherence. We demonstrate how these units can be connected into circuits to perform logic operations (e.g., A OR B OR C) and identify the different ways in which these operations can be initiated and manipulated by top-down feedback. We show that more sophisticated and flexible top-down control is possible when the gain of units is modulated by not only top-down feedback but by oscillatory coherence. Specifically, it is possible to not only add units to, or remove units from, the operation of a higher-level unit using top-down feedback, but it is also possible to modify the type of role that a unit plays in the operation. Finally, we explore how different network properties affect top-down control and processing in large networks. Based on this, we make predictions about the likely connectivities between certain brain regions and relate our findings to those of experimental studies, where neurons in different cortical regions are recorded during goal-directed, behavioral tasks [1].
